# A possible mechanism of horseback riding on dynamic trunk alignment

**DOI:** 10.1016/j.heliyon.2018.e00777

**Published:** 2018-09-11

**Authors:** Ryota Funakoshi, Koji Masuda, Hidehiko Uchiyama, Mitsuaki Ohta

**Affiliations:** Department of Human and Animal-plant Relationships, Graduate School of Agriculture, Tokyo University of Agriculture, 1737, Funako, Atsugi, Kanagawa, Japan

**Keywords:** Biophysics, Rehabilitation, Physiology

## Abstract

The study aimed to clarify the regularity of the motions of horse's back, rider's pelvis and spine associated with improvement of rider's dynamic trunk alignment. The study used a crossover design, with exercise using the horseback riding simulator (simulator hereafter) as the control condition. The experiments were conducted at Tokyo University of Agriculture Bio-therapy Center. The sample consisted of 20 healthy volunteers age 20–23 years. Participants performed 15-min sessions of horseback riding with a Hokkaido Pony and exercise using the simulator in experiments separated by ≥2 weeks. Surface electromyography (EMG) after horseback riding revealed decreased activity in the erector spinae. Exploratory data analysis of acceleration and angular velocity inferred associations between acceleration (Rider's neck/longitudinal axis [Y hereafter]) and angular velocity (Horse saddle/Y) as well as angular velocity (Rider's pelvis/Y) and angular velocity (Horse saddle/Y). Acceleration (Rider's neck/Y) tended to be associated with angular velocity (Rider's pelvis/Y). Surface EMG following exercise revealed decreased activity in the rectus abdominis and erector spinae after the simulator exercise. Horseback riding improved the rider's dynamic trunk alignment with a clear underlying mechanism, which was not observed with the simulator.

## 1 Introduction

The physiological basis of hippotherapy is to transfer the movement of the horse to the body of the patient in a three-dimensional plane [1]. Hippotherapy is based on the theory that a horse guides a repetitive periodic movement pattern (similar to that of human walking) for the riders [2]. In hippotherapy, the patient is a passive element and their body movement adapts to the movement of the horse [3].

Previous research showed that trunk alignment improved in riders who participated in long-term hippotherapy [4, 5] and was thought to be due to the tilt of the pelvis and rotation of the lumbar spine. Subjective observations of a walking person and a person riding a horse have shown rectilinear and rotational motions in the three main axes [6]. However, few reports have clarified the motional regularity of the horse's back and the rider's pelvis and neck, and its association with improving the trunk alignment of the rider. Improvement of trunk alignment leads to a better posture balance [7]. Improvement of posture balance leads to raising the quality of life [8, 9]. It is important to evaluate whether trunk alignment is improved from horseback riding.

The study aimed to clarify the regularity of the motions of horse's back, rider's pelvis and spine associated with improvement of rider's dynamic trunk alignment. We hypothesized that horseback riding improves a rider's dynamic trunk alignment through one or more mechanisms. Surface electromyography (EMG) is a tool that is often used to study muscle activity related to posture and movement [10, 11, 12]. Postural changes are known to affect surface EMG recording [13]. In this study, surface EMG was used to measure the muscle activity which indicates how a rider's dynamic trunk alignment improves from horseback riding. Exploratory data analysis was performed using accelerometers, which is a popular method in the kinesiological field [14, 15, 16]. In this study, we measured acceleration and angular velocity simultaneously, enabling analysis of the rectilinear and rotational motions of the horse and rider to elucidate the aforementioned mechanism.

## 2 Methods

The experiments in this study were approved by The Human Research Ethics Committee (approval number: 1134) and Animal Experiment Ethics Committee (approval number: 260030) at the Tokyo University of Agriculture in accordance with the World Medical Association Declaration of Helsinki. All participants provided verbal informed consent prior to commencement. Participants were not injured by any factors, such as sensors or horseback riding, in this study.

### 2.1 Study design

The study was a crossover design, with exercise using the horseback riding simulator (simulator hereafter) as the control condition. The participants were requested to complete one experiment followed by the second experiment after an interim period of ≥2 weeks.

### 2.2 Participants

Inclusion criteria were as follows: 1) age 20–25 years (both men and women), 2) good general health and experience with horseback riding, 3) experience at the horse riding facilities used in this study. Individuals with a medical history of low back pain were excluded from the study.

All participants were young adults (mean age = 21.5 years, SD = 0.8, range = 20–23). Their heights ranged from 149.4 to 174.3 cm (mean = 161.5, SD = 8.5) and their weights ranged from 38.0 to 65.7 kg (mean = 54.5, SD = 7.9). All participants had body mass index values less than 25 kg/m^2^ (mean = 20.8, SD = 1.5, range = 17.0–23.0). They performed horseback riding a few times during the interim period. However, the participants did not perform horseback riding or other exercises prior to the experiment on the day of the test. All data were collected at the Tokyo University of Agriculture Bio-therapy Center.

### 2.3 Horseback riding

Horseback riding (15 min) was performed on flat riding ground (15.7 m × 32 m). A Hokkaido Pony (8-year-old gelding, withers height of 133.5 cm, body length of 138.7 cm) was used as the test horse in all tests. The horse used in this study had the standard physique for hippotherapy in Japan, with a withers height more than 120 cm to ensure that a rider's feet would not touch the ground. The same saddle (Bruno Delgrange, Partition) was used for the whole experiment. The participants rode without placing their feet in the stirrups. The test horse was led at a constant walking speed (mean 59.3 m/min), which is the speed of a human walking slowly. The mean walking speed of the horse was calculated by placing marks on the four corners of the riding ground and recording the lap time with the stopwatch. The bias may be reduced if the walking speed is strictly controlled [6]; therefore, we kept the speed constant. The test horse first walked clockwise for 7.5 min and then counter-clockwise for 7.5 min.

### 2.4 Simulator exercise

The participants performed the simulator exercise (15 min) in the assessment room of the stable next to the riding ground. The same simulator (EU-JA30, Panasonic Corporation, Osaka, Japan) was used for each experiment. The participants rode without placing their feet in the stirrups. The simulator had five modes (beginner, soft, hard, leg, hip), five different speeds (1–5) within each mode (except beginner mode), and 10 levels of vibration intensity in the system. The simulator was set to soft mode (speed 2), and two to six levels of intensity were mixed throughout the 15-min session.

### 2.5 Outcome measures

**Fig. 1:**
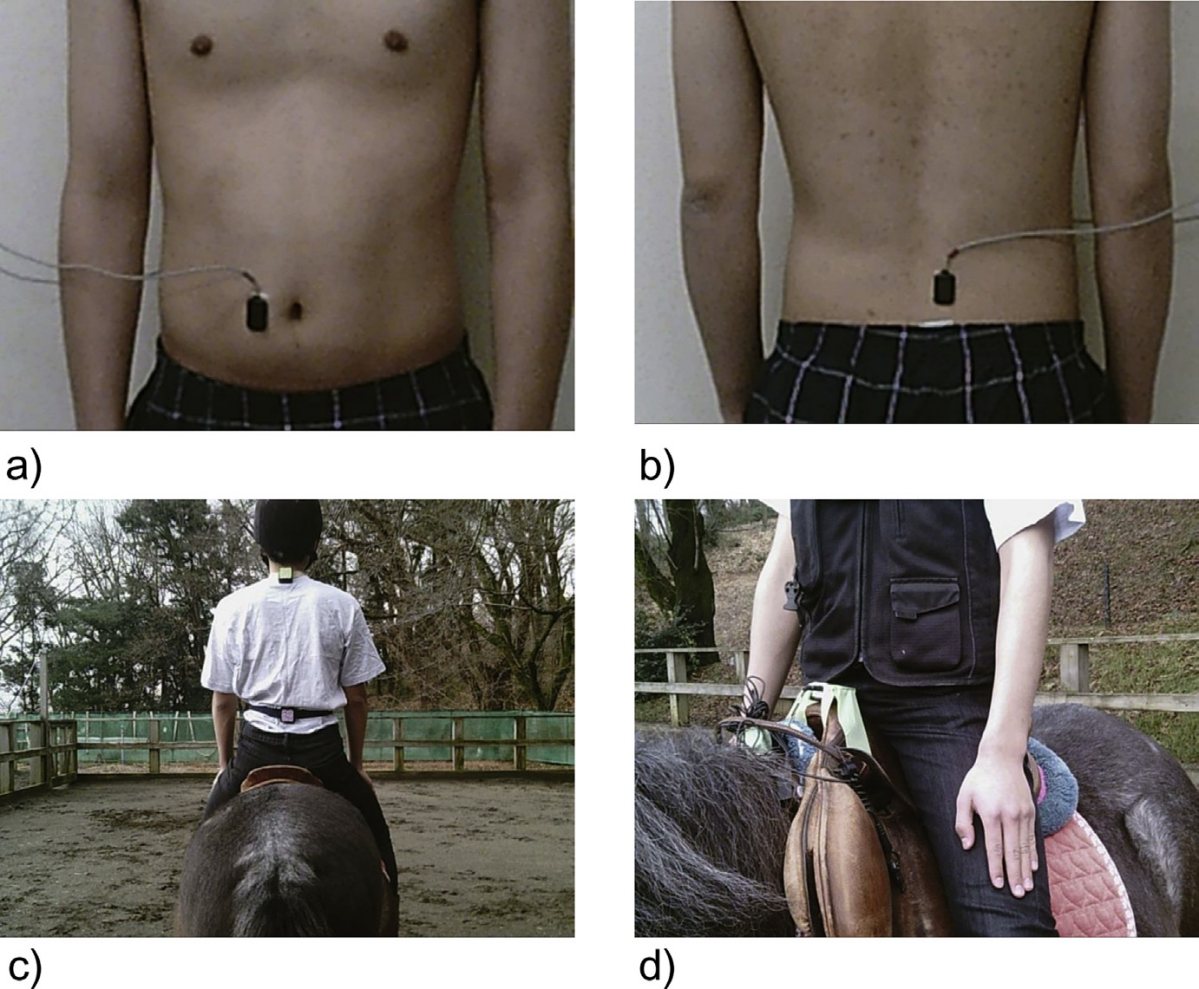
Participant with surface electromyography electrodes and accelerometers. Surface electromyography electrodes were attached to the rectus abdominus (a, 3 cm from the belly button) and the erector spinae (b, at the height of the Jacoby line, 2 cm from the spinous process). Accelerometers were attached to rider's neck (c, vertebra prominens), rider's pelvis (c, Jacoby line), horse saddle (d, pommel).

Surface EMG of participants was measured using a low-power medical telemetry system, WEB-5500 (Nihon Kohden Corporation, Tokyo, Japan). The electrode material was pure silver (Ag), and the dimensions were 28 mm long and 18 mm wide. The interelectrode distance was 10 mm. The measurement position was cleaned with alcohol-impregnated cotton. No skin or hair were removed, and no gel or paste was used with the electrodes. Outcome measures included trunk muscle measurements (rectus abdominis and erector spinae, n = 10, 4 males and 6 females). Trunk muscles (rectus abdominis and erector spinae) were chosen because their activity was easy to measure during walking (Fig. 1a and b). The electrode location and orientation were determined according to the method described by Kizuka et al. [17]. The sampling rate was 2000 Hz. The participants performed treadmill walking (4 km/h for 60 s) using WinFDM-T (zebris Medical GmbH, Isny, Germany) in the assessment room of the stable. The recording was done before and after the exercise. After all the data was analyzed, we carried out the comparisons.

Accelerometer parameters were measured using sensor of MVP-RF8-BC (Microstone corporation, Nagano, Japan). The sensors were attached to vertebra prominens of rider's neck and Jacoby line of rider's pelvis and pommel of the horse saddle (Fig. 1c and d). The total number of sensors used in this experiment was three. Two sensors were attached to the participant by winding the belt just before riding the horse (or simulator). One sensor was fixed to the saddle with tape. The calibration was performed when the horse (or simulator) and rider were not moving after riding. The sensors were removed shortly after exercise. To assess acceleration and angular velocity, 18 items were simultaneously measured along the lateral (X), longitudinal (Y), and vertical axes (Z) (n = 20) during the exercise. A sampling rate of 20 ms, i.e., 50 Hz, was used for recording the acceleration data. We obtained 45000 data points in 15 min for each participant. Data were compared between the horseback riding and simulator exercise.

### 2.6 Statistical analyses

Statistical analyses were performed using add-in software for Excel, Statcel3 (The Publisher OMS Ltd., Tokyo, Japan). A p-value of 0.05 was considered statistically significant for all analyses. Effect sizes were calculated using G*Power, which is a free power analysis program for a variety of statistical tests [18, 19], in post-hoc analyses upon the completion of all experiments. Cohen's d is a measure of the standardized mean difference (difference in group means divided by the pooled standard deviation).

Mean integrated EMG (iEMG, μV·s) was used to compare surface EMG parameters and was calculated as the average rectified value for the duration of three strides (one stride was counted as the duration between two right heel contacts). The mean iEMG was not normalized. The parameter evaluated was only the iEMG. The comparisons before and after horseback riding (HR) and exercise on the riding simulator (ES) were done with the Wilcoxon signed-rank test.

Acceleration was converted into root mean square (A-rms). Angular velocity was converted into mean absolute value (AV-mav). The Wilcoxon signed-rank test was chosen to analyze accelerometer parameters. All nine acceleration measurements for each of the 20 participants were processed using the fast Fourier transform algorithm to create frequency data for the 20 participants, which was processed to calculate the mean for each item. The mean frequency data of the 20 participants was processed by spectral analysis of each item using the graphing software OriginPro 8.5 (OriginLab Corporation, Massachusetts, USA). The maximum peak frequency was calculated using spectral analysis for each item.

For the exploratory data analysis, the Spearman's rank correlation coefficient was used to analyze the relationship of accelerometer measurements between rider's neck and horse saddle, rider's pelvis and horse saddle, and rider's neck and rider's pelvis. Multiple regression analysis was performed when two or more accelerometer parameters showed a significant correlation.

## 3 Results

### 3.1 Surface EMG parameters

In this category, data were analyzed in 10 participants who underwent measurement of the trunk muscles (Table 1).

**Table 1:** Change in surface electromyography parameters before and after the exercise with HR/ES.

	HR						ES					
		Pre		Post		Effect Size Cohen's d		Pre		Post		Effect Size Cohen's d
		Mean	SD	Mean	SD			Mean	SD	Mean	SD	
Rectus abdominis		36.4	20.0	25.9	26.1	0.34	*	28.5	11.9	16.2	4.7	1.06
Erector spinae	**	94.3	102.0	30.7	6.4	0.61	**	93.2	97.8	27.6	7.7	0.70

Values (iEMG, μV·s) expressed as mean ± SD. Rectus abdominis and erector spinae, n = 10. ^∗^ and ^∗∗^ Statistically significant difference between pre and post tests (P < .05, P < .01, Wilcoxon signed-rank test). Abbreviations: HR, horseback riding; ES, exercise using the horse riding simulator.

The surface EMG activity significantly decreased in the erector spinae with horseback riding (P < .01), and in the rectus abdominis (P < .05) and erector spinae (P < .01) with the simulator exercise.

### 3.2 Accelerometer parameters

The data from all 20 participants were analyzed (Tables 2 and 3). Maximum peak frequency and most parameters of acceleration and angular velocity were significantly greater for horseback riding (HR) than for exercise on the horse riding simulator (ES) (Tables 2 and 3). The acceleration (X and Z axis of Horse saddle in A-rms) and the angular velocity (Y and Z axis of Horse saddle in AV-mav) in exercise on the simulator were smaller than those in horseback riding.

**Table 2:** Comparison of accelerometer parameters between horseback riding and exercise using the simulator.

				HR		ES		Effect Size Cohen's d
				Mean	SD	Mean	SD	
A-rms (m/s^2^)	Rider's neck	X		0.98	0.28	0.86	0.24	0.31
		Y	**	1.24	0.74	0.88	0.32	0.56
		Z	**	1.45	0.43	0.66	0.24	1.69
	Rider's pelvis	X	*	0.77	0.17	0.65	0.17	0.49
		Y		1.34	0.56	1.42	0.52	0.12
		Z	**	1.42	0.22	0.63	0.17	3.57
	Horse saddle	X	**	1.09	0.20	0.80	0.07	1.30
		Y		1.31	0.18	1.29	0.09	0.13
		Z	**	0.95	0.09	0.41	0.06	4.84
AV-mav (deg/s)	Rider's neck	X	*	18.55	6.15	22.66	5.27	0.56
		Y	**	12.30	1.62	10.11	3.07	0.66
		Z	*	9.88	0.94	8.29	2.47	0.70
	Rider's pelvis	X		17.81	5.71	14.94	3.41	0.46
		Y		10.43	3.48	10.96	3.33	0.10
		Z		16.45	4.09	14.16	4.38	0.35
	Horse saddle	X		12.51	5.57	10.80	1.37	0.31
		Y	**	9.32	1.54	7.45	0.50	1.21
		Z	**	11.39	4.67	4.58	1.53	1.32

Values expressed as mean ± SD. HR and ES, n = 20. ^∗^ and ^∗∗^ Statistically significant difference between HR and ES (P < .05, P < .01, Wilcoxon signed-rank test). Abbreviations: HR, horseback riding; ES, exercise using the horse riding simulator; A-rms, acceleration converted into root mean square; AV-mav, angular velocity converted into mean absolute value; X, lateral axis; Y, longitudinal axis; Z, vertical axis.

**Table 3:** Maximum peak frequency in horseback riding and exercise using the simulator.

		Maximum peak frequency (Hz)	
		HR	ES
Rider's neck	X	0.83	0.37
	Y	1.63	0.73
	Z	1.66	0.73
Rider's pelvis	X	0.78	0.37
	Y	1.64	0.73
	Z	1.66	0.73
Horse saddle	X	0.82	0.37
	Y	1.64	0.73
	Z	1.66	0.89

HR and ES, n = 20. Abbreviations: HR, horseback riding; ES, exercise using the horse riding simulator. X, lateral axis; Y, longitudinal axis; Z, vertical axis.

### 3.3 Exploratory data analysis

**Fig. 2:**
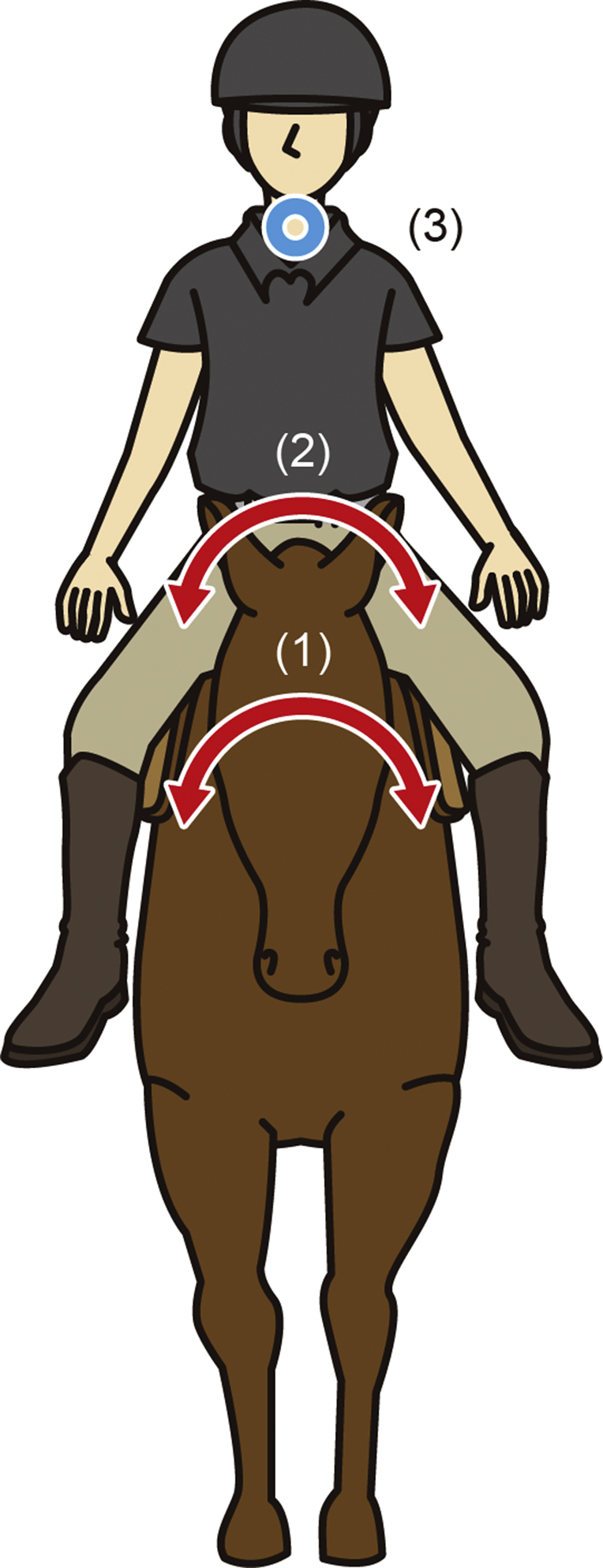
Motion of the rider during horseback riding. Blue point: Acceleration, Red arrow: Angular velocity. (1) to (2): the rotational motion in the Y axis generated by the horse led to lateral tilt of the rider's pelvis. (2) to (3): lateral tilt of the rider's pelvis produced spinal rotation and bending, and consequently, movement in the longitudinal direction of the rider's neck.

In this category, data were analyzed in all 20 participants (Table 4 and Fig. 2).

**Table 4:** Correlation and multiple regression analysis between accelerometer parameters in horseback riding as exploratory data analysis.

Model	Dependent variable	Independent variable		Correlation		Multiple regression analysis			
				rs	P	β	R^2^	F	P
1	A-rms (Rider's neck/Y)	A-rms (Horse saddle)	X	0.29	n.s.	-			
			Y	0.25	n.s.	-			
			Z	0.21	n.s.	-	0.58	11.85	**
		AV-mav (Horse saddle)	X	0.07	n.s.	-			
			Y	0.70	**	0.77			
			Z	−0.52	*	0.01			
2	AV-mav (Rider's pelvis/Y)	A-rms (Horse saddle)	X	0.03	n.s.	-			
			Y	0.59	**	0.20			
			Z	0.41	n.s.	-			
		AV-mav (Horse saddle)	X	0.44	n.s.	-	0.54	9.82	**
			Y	0.71	**	0.57			
			Z	−0.16	n.s.	-			
3	A-rms (Rider's neck/Y)	A-rms (Rider's pelvis)	X	−0.08	n.s.	-			
			Y	0.20	n.s.	-			
			Z	0.44	n.s.	-	0.26	2.92	n.s. (0.08)
		AV-mav (Rider's pelvis)	X	0.53	*	-0.09			
			Y	0.57	*	0.56			
			Z	0.45	n.s.	-			

Values expressed as rs, β, R^2^, F. n = 20. ^∗^ and ^∗∗^ Statistically significant (P < .05, P < .01, Spearman's rank correlation coefficient). Abbreviations: HR, horseback riding; ES, exercise using the horse riding simulator; A-rms, acceleration converted into root mean square; AV-mav, angular velocity converted into mean absolute value; X, lateral axis; Y, longitudinal axis; Z, vertical axis.

In model 1, A-rms (Rider's neck/Y) was positively correlated with AV-mav (Horse saddle/Y) (rs = 0.70, p < 0.01). A-rms (Rider's neck/Y) was negatively correlated with AV-mav (Horse saddle/Z) (rs = −0.52, p < 0.05). Multiple regression analysis of these three variables revealed A-rms (Rider's neck/Y) was related to AV-mav (Horse saddle/Y) (β = 0.77, p < 0.01, R^2^ = 0.58, F = 11.85).

In model 2, AV-mav (Rider's pelvis/Y) was positively correlated with A-rms (Horse saddle/Y) (rs = 0.59, p < 0.01), and AV-mav (Horse saddle/Y) (rs = 0.71, p < 0.01). Multiple regression analysis of these three variables revealed AV-mav (Rider's pelvis/Y) was related to AV-mav (Horse saddle/Y) (β = 0.57, p < 0.01, R^2^ = 0.54, F = 9.82).

In model 3, A-rms (Rider's neck/Y) was positively correlated with AV-mav (Rider's pelvis/X) (rs = 0.53, p < 0.05) and AV-mav (Rider's pelvis/Y) (rs = 0.57, p < 0.05). Multiple regression analysis of these three variables revealed A-rms (Rider's neck/Y) tended to be related to AV-mav (Rider's pelvis/Y) (β = 0.56, p = 0.08, R^2^ = 0.26, F = 2.92), although the association was not significant.

The above correlations were not observed with the simulator exercise.

## 4 Discussion

Our hypothesis that horseback riding improves a rider's dynamic trunk alignment through one or more mechanisms could be accepted in this study.

In the horseback riding, the movement of pelvis and spine of the riders was affected by the movement of the horse (Table 4, Fig. 2). In this mechanism, the rotational motion generated by the horse in the Y axis led to lateral tilt of the rider's pelvis, which indicated spinal rotation and bending in the rider that caused longitudinal movement of the rider's neck. The lateral shift of the rider's lumbar spine is associated with the horse's trunk movement [20]. The lateral movement of the horse's abdomen causes displacement in the lateral direction, while the pelvic movement of the rider continues [3]. Generally, when the leftward and the rightward accelerations alternate, forces to turn left and right are generated. We considered our results to be consistent with previous research. Pelvis of the riders absorbed the strong rectilinear motion and the large rotational motion at the saddle (Table 2). At the same time, we inferred that the neck of the rider rotated substantially to maintain their posture on the horse. Thus, the movement of the rider's body during horseback riding may improve dynamic trunk alignment.

The frequency peak of human walking matches that of horse walking in the lateral (2.5–3.0 Hz), longitudinal (1.5–2.0 and 3.5–4.0 Hz), and vertical axes (1.5–2.0 Hz) [21]. The results of this study were roughly the same (Table 3). The movement related to the improvement of the dynamic trunk alignment described above was performed with the same rhythm as human walking. We assume that each person has a central pattern generator (CPG) which generates walking rhythms [22]. If CPG function declines due to some factor such as aging or disease, horseback riding may be an effective exercise.

Horses have four types of steps (the walk, trot, canter, and gallop). The walk is the slowest step. The walk creates a four beat rhythm, with the left hind leg, left foreleg, right hind leg, and right foreleg landing in succession. The trot occurs at a higher speed than the walk. The trot involves two beats, with two diagonally opposite limbs landing at the same time. These steps (the walk and the trot) are the main ones used in hippotherapy.

The movement through space (moving forward continuously) is the primary characteristic of riding real horses. In the walk, when the left hind leg of the horse is landing, the rider's neck accelerates slightly depending on the acceleration of the horse itself. When the left foreleg of the horse is landing, the rider's pelvis tilts to left, and the rider's neck decelerates slightly depending on the deceleration of the horse itself. When the right hind leg of the horse is landing, the rider's neck accelerates slightly depending on the acceleration of the horse itself. When the right foreleg of the horse is landing, the rider's pelvis tilts to right, and the rider's neck decelerates slightly depending on the deceleration of the horse itself. The simulator moves the pelvis in a backward and forward (and lateral) cycle, but does not move through space since the simulator is sitting stationary on a floor. When the simulator tilts forward, the rider's pelvis tilts forward. At the same time, the rider's neck moves backward slightly. When the simulator becomes horizontal, the rider's pelvis tilts backward. At the same time, the rider's neck moves forward slightly.

The exercise effect did not differ between horseback riding and the simulator exercise in this study (Table 1). Walking muscle activity of participants decreased after horseback riding and simulator exercise. One of the reasoning behind applying hippotherapy is to increase mobility and also engage different necessary muscles in a rhythmical pattern which could improve the overall movement pattern of patients with movement disorders. Adapting to the movements of horse involves changes in muscle utilization and joint movements which may lead to increase adductor muscle activity [10]. Although muscle activity is increase in hippotherapy, people without physical impairment have adapted to the movement of the horse, controlling their muscle activity during horseback riding [12]. In this study, there is a possibility that the adductor muscle of the lower limb was greatly used during horseback riding (or simulator). Whereas, trunk muscle activity was controlled as a result of adaptating to the movements of horse (or simulator). The dynamic trunk alignment of the rider was improved, the tension of trunk muscles was relieved and the amount of muscle activity decreased. The low muscle activity level is observed in the flat posture, which is similar to slumped posture, with the least muscle activity [13]. We interpret these results as indicating that participants' dynamic trunk alignment while riding had positive effect after both exercise conditions. The acceleration (X and Z axis) and the angular velocity (Y and Z axis) of the simulator were smaller than horseback riding (Table 2). The acceleration on the Y axis and the angular velocity on the X axis were the same in horseback riding and the simulator (these two are interpreted as the intensity in the ‘longitudinal direction’). In this study, the simulator worked to move the pelvis in a back and forth (and lateral) cycle slowly, creating an immediate effect. Therefore, our study clearly demonstrated that the rider motions from the horseback riding more improved the dynamic trunk alignment of the rider than from the simulator.

Interventional studies have shown the efficacy of hippotherapy on muscle activity, functional ability, gait parameters, and postural stability in cerebral palsy [10, 23], multiple sclerosis [24, 25], down syndrome [26, 27], and gerontological science [28, 29]. Whereas, few reports described the motion mechanism of horseback riding which justify the efficacy on the dynamic trunk alignment. We sure that the findings of this study is the possible mechanism which improve knowledge on the benefits of horseback riding.

We expect future studies to improve on the present study in two ways. First, the movement through space is the primary difference between riding a real horse and riding a simulator. Measurements of EMG on the rider's neck may indicate differences in muscle activity between riding a real horse and riding a simulator. Second, only healthy individuals participated in this study. This study advanced understanding of the basic mechanisms of horseback riding. Hippotherapy may be useful in treating patients with vertigo and other conditions.

## 5 Conclusion

Horseback riding improved the rider's dynamic trunk alignment with a clear underlying mechanism (the rotational motion generated by the horse in the longitudinal axis led to lateral tilt of the rider's pelvis, which indicated spinal rotation and bending in the rider that caused longitudinal movement of the rider's neck), although this mechanism was not seen in exercise using the simulator. Horseback riding can adjust a rider's balance of the whole body around the spinal column. Therefore, it might be usefully applied in the treatment of cerebral palsy and other conditions.

## Declarations

### Author contribution statement

Ryota Funakoshi: Conceived and designed the experiments; Performed the experiments; Analyzed and interpreted the data; Wrote the paper.

Koji Masuda: Analyzed and interpreted the data.

Hidehiko Uchiyama: Conceived and designed the experiments; Analyzed and interpreted the data; Wrote the paper.

Mitsuaki Ohta: Analyzed and interpreted the data; Wrote the paper.

### Funding statement

This research did not receive any specific grant from funding agencies in the public, commercial, or not-for-profit sectors.

### Competing interest statement

The authors declare no conflict of interest.

### Additional information

No additional information is available for this paper.
